# The social determinants of adolescent anxiety and depression in peri-urban South Africa

**DOI:** 10.1371/journal.pmen.0000173

**Published:** 2024-11-21

**Authors:** Laura Rossouw, Kathryn Watt, Leslie L. Davidson, Chris Desmond

**Affiliations:** 1 Health Economics and Epidemiology Research Office, Faculty of Health Sciences, University of the Witwatersrand, Johannesburg, Gauteng, South Africa; 2 Centre for Rural Health, School of Nursing and Public Health, University of KwaZulu-Natal, Durban, KwaZulu-Natal, South Africa; 3 Department of Epidemiology, Mailman School of Public Health, Columbia University, New York, New York, United States of America; 4 Department of Pediatrics, Columbia College of Physicians and Surgeons, Columbia University, New York, New York, United States of America; 5 School of Economics and Finance, University of the Witwatersrand, Johannesburg, Gauteng, South Africa

## Abstract

The environment in which adolescents live impacts their mental health, through social determinants. We examine the impact of social determinants on anxiety, and depression in adolescents (aged 13–19) in peri-urban KwaZulu-Natal, South Africa. Using structural equation modelling, we identify direct relationships between social determinants and depression/anxiety, as well as indirect pathways between social determinants. Our findings indicate that living with the biological mother, the perception of family and peer support, school enrolment, and a positive sense of school membership may protect adolescent’s mental health. Conversely, exposure to community violence may be directly detrimental to adolescent mental health and indirectly harm mental health through the negation of a sense of school membership. Understanding the potential direct and indirect pathways between social determinants and adolescents’ mental health in resource-constrained contexts can inform interventions to protect young people’s well-being through the identification of appropriate entry points closer to and further from the adolescent and their household.

## 1. Introduction

Adolescence, with its myriad of physical, neurobiological, hormonal and socio-emotional changes, is a period in which mental health disorders often begin [1]. Unlike physical illness, mental illness is the scourge of youth. Most mental health disorders begin at 15 years of age and two-thirds to three-quarters are manifest before the age of 25 [2]. Mental health disorders are prevalent among adolescents worldwide- in 2019, it was estimated that 28% of individuals aged 10–19 globally experienced a mental disorder [3]. Without treatment, mental health disorders emerging in adolescence can have dire effects beyond this period including death, disability, loss of human potential and productivity [2]. Given the lifelong impacts adolescent mental disorders can have it is arguably better to invest in preventive measures rather than responding to disorders after their onset [4].

The economic and social conditions in which adolescents live impact their physical and mental health [5]. These social determinants of health and mental health include characteristics of adolescents’ structural environment, such as access to adequate nutrition, healthcare and education, their material circumstances, and their exposure to adverse events such as experiencing violence or discrimination [6]. Adolescents’ unique developmental vulnerabilities mean they are influenced by social determinants differently to children and adults. Their brain development makes them particularly vulnerable to negative environmental influences: for example, they are more likely to engage in positive and negative risk-taking, and sensation- and social status-seeking behaviours [7, 8].

This paper assesses the contribution of social determinants to the mental health of adolescents in a resource-constrained setting in KwaZulu-Natal, South Africa. The common mental disorders (CMD), anxiety, and depression highly prevalent among adolescents globally, are prevalent in South Africa. Studies have highlighted the severity of this issue within the country. In 2011, 25% of high school learners in South Africa between grade 8 and grade 11 felt so sad or hopeless in the prior six months that they stopped engaging in their usual activities for two or more consecutive weeks, while 18% had attempted to end their lives on one or more occasions [9]. In South Africa, poor adolescent mental health is linked to lower levels of school completion, later formalised employment [10], and health-related disability [11].

In KwaZulu-Natal, many children and adolescents face multiple socio-economic challenges. In 2022, 24.8% of children in KwaZulu-Natal were living with neither biological parent, 22.9% of households experienced food inadequacy, and 46.7% of 15-64-year-olds were unemployed including discouraged work seekers [12, 13]. In 2017, the HIV prevalence among youth aged 15–24 in KwaZulu-Natal was 9% [14]. These socio-economic challenges in combination with the prevalence of CMDs in young South Africans, highlight social determinants as an important entry point for policymakers and practitioners attempting to protect young people’s mental health.

Our analysis is informed by the WHO’s Conceptual Framework of the Social-Determinants of Health and Viner et al’s interpretation and application of this framework to adolescent health [5, 15]. We apply this approach to a single wave of data from an adolescent cohort study using structural equation modelling to test possible direct and indirect relationships between selected social determinants and depression and anxiety.

## 2. Conceptual framework

The WHO Conceptual Framework of the Social-Determinants of Health outlines the contribution of social and economic factors to health outcomes and health inequalities, and the effect of the interaction between determinants on health outcomes [16]. Key to the framework, and our approach, is the distinction between social determinants which are (1) structural (systems and opportunities), and those that are (2) proximal (the circumstances and experiences of daily life). This socio-ecological framing of social determinants allows us to consider the interactions of influences closer to and further from the adolescent and their household.

Structural determinants of mental health are the socio-economic conditions in which a person lives that influence their well-being. These include governance and political systems, macroeconomic and social policies, socio-economic status, as well as cultural norms and social institutions.

Proximal determinants represent the immediate circumstances of daily life, such as housing, relationships, community exposures, and social support. Proximal determinants are the pathways through which structural determinants impact physical and mental health; for example food security is a proximal determinant which often stems from the structural determinants socio-economic status and food environment.

Notably this paper focuses on these structural and proximal determinants only in adolescence; we do not consider earlier life exposures. We used Viner et al.’s adaption of the WHO framework for adolescent health to guide the selection of social determinants from those present in the Asenze Wave 3 data. for inclusion in our model. We situated the selected variables within the literature on the social determinants of adolescent mental health (see Table 1).

## 3. Materials and methods

### 3.1. Data and variables

#### 3.1.1. The Asenze study.

We use data from wave 3 of the Asenze study, a population-based longitudinal cohort study begun in 2008 in The Valley of a Thousand Hills, a peri-urban area of KwaZulu-Natal province. Four Waves of data collection have been undertaken: Waves 1 and 2 focused on child neurodevelopment, cognitive function, child behavioural problems, and physical health, while Waves 3 and 4 of the cohort study focused on identifying risk and protective factors for HIV acquisition, risky sexual behaviour, substance use, school dropout, neurocognitive difficulties, and other poor outcomes in adolescence. In 2008, 2,049 eligible children within the study age range living in 1,818 households and looked after by 1,893 primary caregivers were invited to participate in the study through door-to-door recruitment. Ultimately, 1581 children aged 4–6 years, along with their primary caregiver, were enrolled in Wave 1. In 2010, Wave 2 had a 90% retention rate enrolling 1409 children aged 608 and their caregivers. Wave 3 commenced in October 2019, enrolling 1176 adolescents aged 13–19 (83% retention) and their primary caregivers. Data collection paused March-August 2020 during the Covid-19 pandemic, resumed in September 2020, and was completed in January 2022. were enrolled. Wave 4 was conducted telephonically in 2022, enrolling 1120 adolescents (95% retention) without their caregivers. In Wave 3 adolescents self-reported on a wide range of domains, including mental health, substance use, sexual risk behaviour, education, access to health care, and peer and family support. Caregivers reported on household socio-economic status, family structure and functioning, and personal well-being, among other areas. In this paper, we select variables from these adolescent and caregiver domains guided by our conceptual framework; the selected variables are detailed in Table 2. For a full description of the cohort see Desmond et al. [45].

#### 3.1.2. Mental health outcomes: Anxiety and depression.

Anxiety was captured using the Generalized Anxiety Disorder 7-item (GAD-7) questionnaire [46]. Adolescents indicated how often, over the last two weeks, they were bothered by feelings such as nervousness, restlessness, worry, annoyance, and dread. Depression was captured using the 9-item Patient Health Questionnaire, modified for adolescents (PHQ-A) [47]. Adolescents indicated how often they felt the symptoms of depression in the prior two weeks. As opposed to using additive methods and cut-offs to calculate a depression and anxiety score, we relied on the latent component aspect of the structural equation model (described under Empirical Strategy).

#### 3.1.3. Social determinants of adolescent mental health.

The social determinants included in our model, as derived from the conceptual framework in Table 1, are described in Table 2.

### 3.2. Empirical strategy

#### 3.2.1. Structural equation modelling.

We use structural equation modelling (SEM) to investigate the relationships between social determinants and mental health outcomes. SEM is useful here because it allows us to test for direct and indirect associations between social determinants and mental health outcomes. Social determinants shape adolescents’ context, for example, their household’s economic wellbeing. This could directly influence their mental health, but it may indirectly influence it via school enrolment, if it leads to dropout because of inability to pay school fees. SEM allows us to specify the indirect pathways we believe may be operating and test if the data fits with that belief. As the data are observational, we cannot say if these associations are causal or not, only if they are there or not. In addition, the SEM allows us to treat anxiety and depression as latent variables, which allows for better estimation and control for measurement error [52].

**Fig 1.**
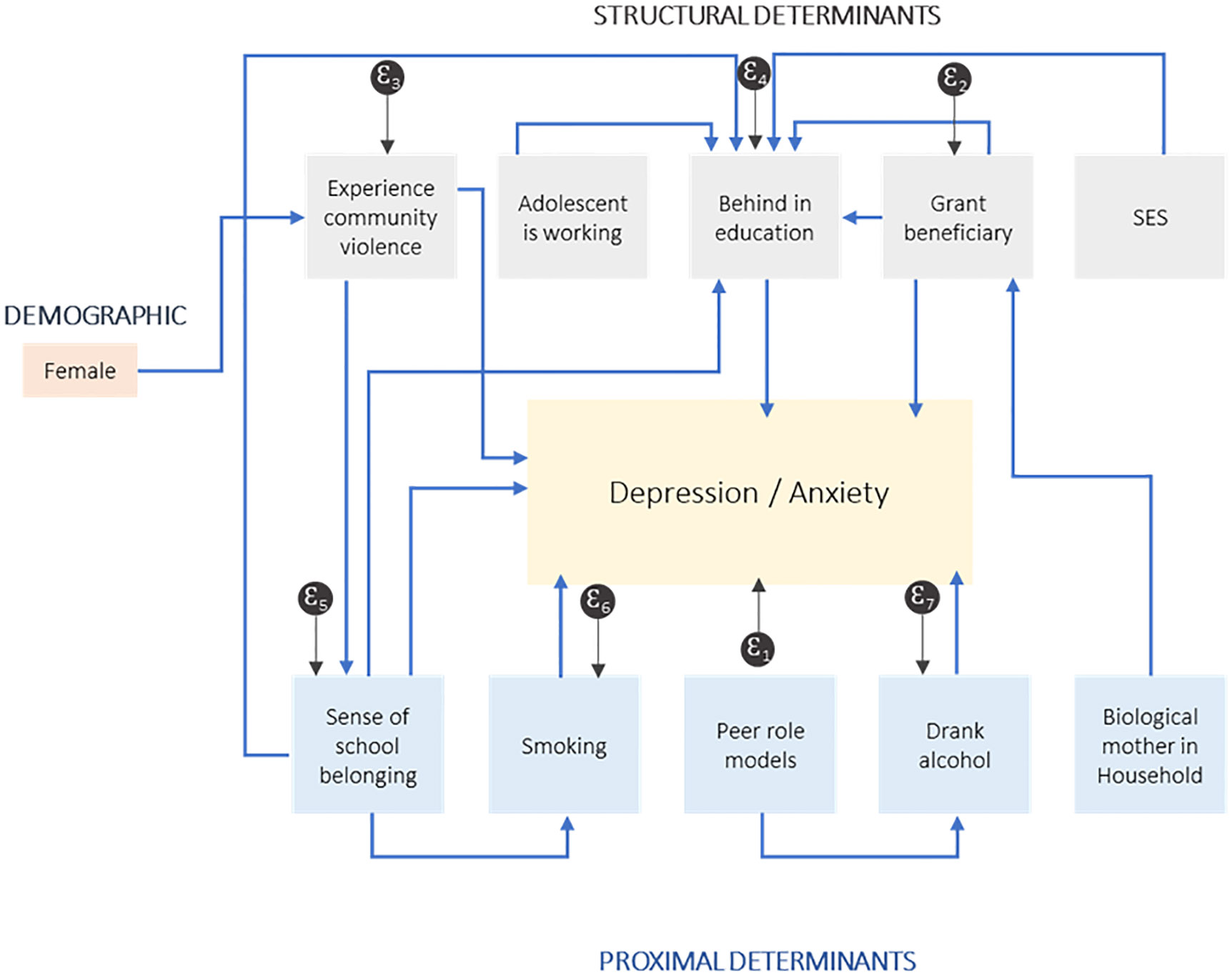
Path analysis and mediators of the social determinants of mental health outcomes.

Possible indirect relationships are derived from the literature (Table 1) and tested empirically, as reflected in Fig 1, and described in Table 3.

SEM models can be tested using many estimation procedures. We use the maximum likelihood estimator with the missing value option to ensure that we do not listwise delete missing observations. Maximum likelihood, an iterative process, estimates the degree to which the model predicts the values of the sample covariance matrix and is robust to non-normal data distribution [53]. Standardized coefficients were reported as effect sizes. All results are reported for statistical significance at a 1%, 5% and 10% level. Stata 16 is used to conduct the analysis.

#### 3.2.2. Anxiety and depression as latent variables.

A useful component of SEM is that unobservable, latent variables can be constructed based on the relationships between observed or manifest variables. Each manifest or observable variable is related to the latent variable through a linear equation. This component of SEM is often used to capture complex relationships such as attitudes, or concepts that cannot be directly observed and measured, but which can be inferred from a set of observed variables. Mental health outcomes are also occasionally analysed as latent concepts using SEM [54, 55]. This allows us to capture the multi-faceted nature of the underlying relationship while also accounting for measurement error, thereby giving a more accurate interpretation of the outcomes [56].

#### 3.2.3. Model fit.

The fit of each model is assessed using the root square mean error of approximation (RMSEA) and the comparative fit index (CFI). Our RMSEAs are 0.05 for the depression model and 0.05 for the anxiety model. These values are equal to 0.05, and so the model is considered a good fit, according to generally accepted guidelines for RMSEA. The CFI ranges from 0 to 1, with a higher value indicating a better fit. The CFIs of our models are 0.756 for Depression and 0.733 for Anxiety.

#### 3.2.4. Sensitivity analysis.

To test their robustness, we replicated our results with different outcome variables. Firstly, rather than treating depression and anxiety as latent outcome variables, we created a binary variable equal to one if adolescents showed any signs of depression or anxiety. The depression outcome variable was valued as one if an adolescent’s PHQ-9 score was between “mild” and “severe”, and anxiety outcome variable valued as one if an adolescent’s GAD-7 score was between “mild and “severe”. The results are largely robust to the change in specification. When considering the binary outcome variable, the female and education variables lose significance.

Secondly, we tested the social determinants model at the extremes, by creating a binary variable equal to one if an adolescent experienced suicidal ideation confirmed by the project’s counselling psychologist. Within the sample of 15.38% adolescents who reported confirmed suicidal ideation, all structural determinants lost significance. Conversely, proximal determinants that were protective of suicidal ideation remained significantly related: sense of school belonging, having pro-social peers, and living with your biological mother. Adolescents experiencing suicidal ideation were much more likely to have smoked in the last 30 days than their peers.

### 3.3. Ethical statement

Asenze wave 3 was reviewed and approved by the University of KwaZulu-Natal Biomedical Research Ethics Committee (UKZN BREC) reference number BE608/18 and by the Columbia University of New York ethics committee (Institutional Review Board) reference number AAAC2559. All participants provided informed consent or assent for all measures reported. Participants had the option to withdraw from the study at any point.

## 4. Results

### 4.1. Descriptive statistics

**Fig 2.**
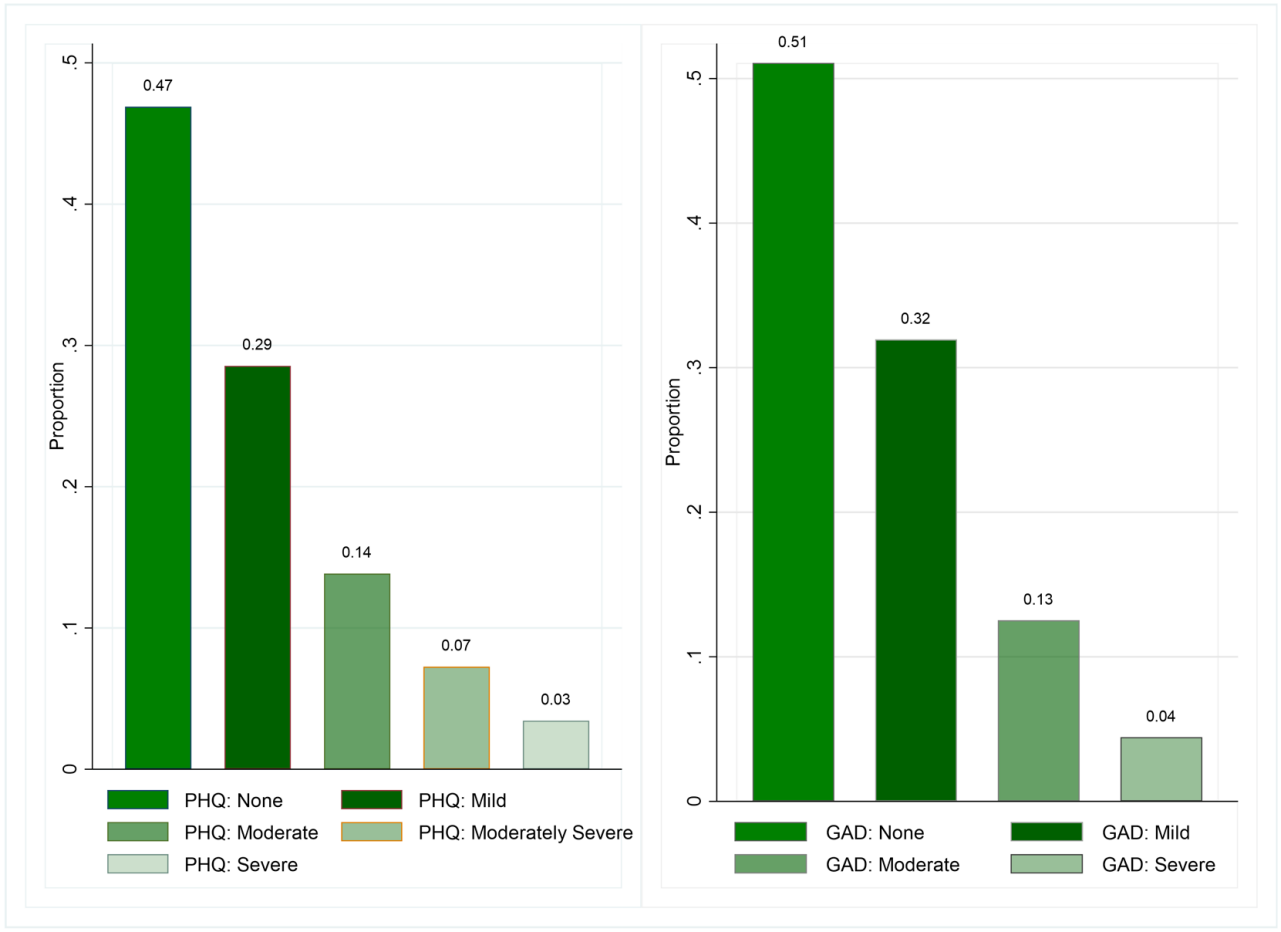
Distribution of Overall PHQ Depression (left) and GAD Anxiety (right) scores.

The overall distribution of the PHQ-9 and GAD-7 scores among the sample adolescents are displayed in Fig 2. Almost half of the adolescents show no symptoms of depression or anxiety. Approximately a quarter of adolescents (24%) show symptoms of moderate to severe depression, while 17% of adolescents show symptoms of moderate to severe anxiety. While the PHQ-9 and the GAD-7 use different outcome scales and questions, there is some overlap in adolescents who show signs of depression and anxiety.

The mean values of the social determinants of adolescent mental health are displayed in Table 4. The sample is on average 15.9 years of age, split evenly by sex. Most caregivers in the sample are female. For 60% of the sample, the household head is unemployed. 85% adolescents benefit from some form of a grant. About 20% of our sample is behind in education, 15% are doing some work for money, and 3.2% are NEET. Almost 71% of adolescents lived with their biological mother, 28% lived with both biological parents. 17% of primary caregivers were the adolescent’s grandparent.

### 4.2. Direct relationship between social determinants and anxiety and depression

Table 5 shows that demographic, structural and proximal social determinants are directly related to adolescent mental health outcomes. Of the demographic determinants, being a female adolescent is significantly associated with a higher probability of both depression (0.175***) and anxiety (0.107***).

Of the structural determinants, falling behind in education (Depression = 0.0999**, Anxiety = 0.0826*), and a higher exposure to community violence (Depression = 0.0229***, Anxiety = 0.0197***), are significantly and positively associated with depression and anxiety. Although a small group, adolescents who are NEET are highly likely to show more signs of anxiety and depression (Depression = 0.418***, Anxiety = 0.255**) than their non-NEET peers.

Several proximal determinants are correlated to adolescents’ mental health. Whether an adolescent’ biological mother lives in their household (Depression = −0.109**, Anxiety = −0.109**), whether they feel supported by friends and peers (Y-HAPP) (Depression = −0.0435***), and whether they feel a sense of school belonging (PSSM) (Depression = −0.0214***, Anxiety = −0.0255***), play a protective role on their mental health outcomes. Adolescents who self-reported smoking cigarettes in the last 30 days are more likely to report being depressed (Depression = 0.172**).

### 4.3. Indirect relationship between social determinants and anxiety and depression

**Fig 3.**
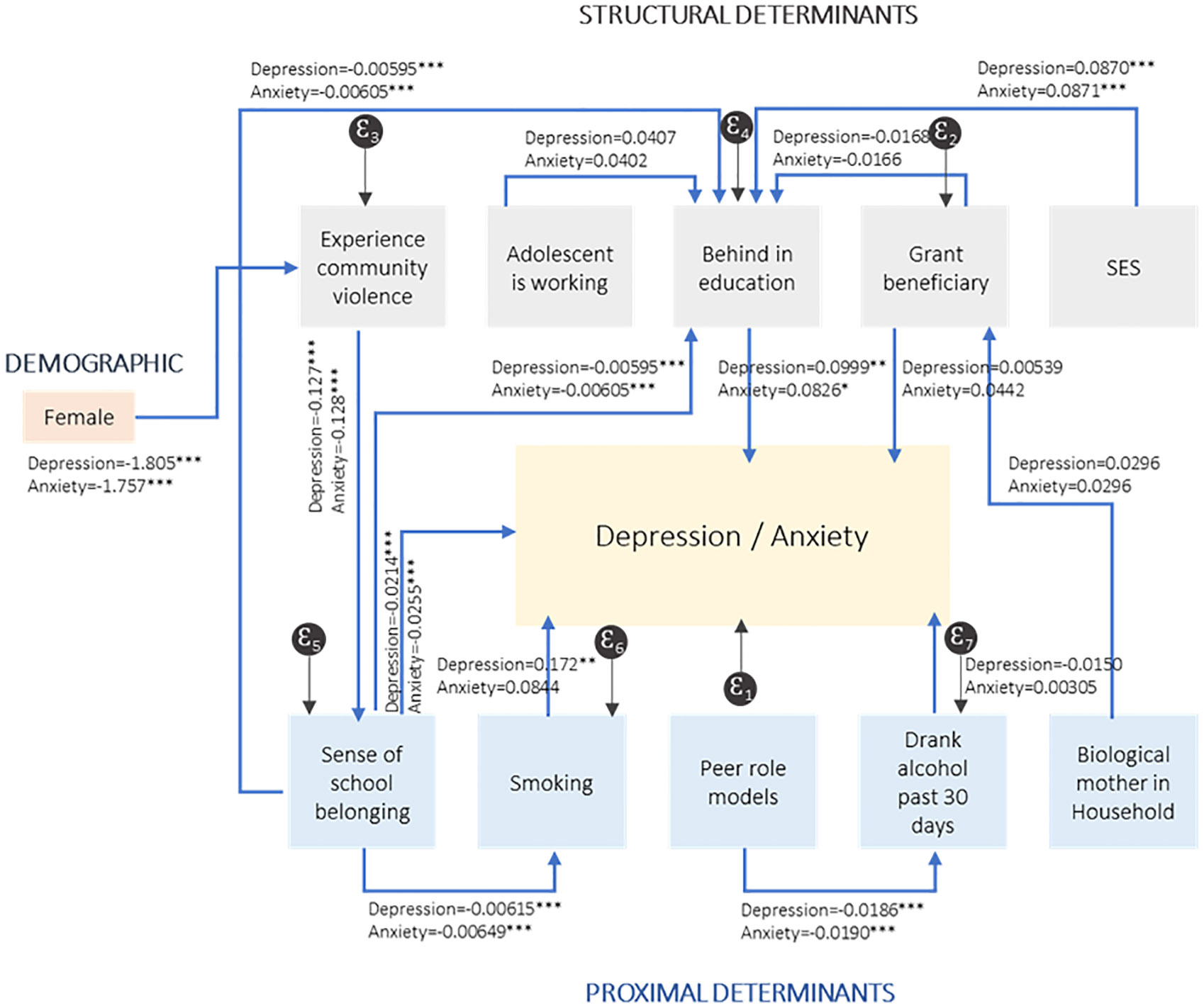
Indirect effects of social determinants on anxiety and depression.

Table 6 reports the indirect effects of social determinants, for both depression and anxiety which should be interpreted in conjunction with the direct effects as reflected in Fig 3.

Several determinants have both direct and indirect effects on mental health outcomes. Adolescents exposed to higher levels of community violence were more likely to experience symptoms of anxiety and depression. From Table 6 and Fig 3, we deduce that exposure to community violence also impacts adolescents’ mental health through its negative effect on school belonging (PSSM) (indirect relationship (e) (−0.127*** for the Depression SEM, and −0.128*** for the Anxiety SEM). We observe a significant and inverted relationship between school belonging and depression (−0.0214***) and anxiety (−0.0255***).

Determinants with both direct and indirect relationships may not be unidirectional. The indirect results also show that sex may be a significant modifier of the relationship between community violence and mental health outcomes (indirect relationship (j)). There is a direct and positive relationship between being female and experiencing poor mental health. However, the results from Fig 3 show that female adolescents are significantly less likely to experience community violence (−1.805*** for the Depression SEM, and −1.757*** for the Anxiety SEM). Therefore, while being a female adolescent is positively linked to poor mental health, this is not through the pathway of exposure to community violence.

We observe several significant relationships between the social determinants themselves, even though these do not necessarily affect mental health outcomes. For instance, exposure to community violence may increase the probability of falling behind in education (indirect relationship (g)). Exposure to community violence has a negative impact on school belonging (−0.127*** for the Depression SEM, and −0.128*** for the Anxiety SEM), while school belonging increases the probability of not falling behind in education (−0.00595*** for the Depression SEM, and −0.00605*** for the Anxiety SEM). Overall, an increased exposure to community violence is indirectly related to an increased probability of falling behind in education.

In Fig 3, we observe that adolescents who experience a sense of school belonging are less likely to have smoked in the last 30 days (−0.00615*** for the Depression SEM, and −0.00649*** for the Anxiety SEM). And adolescents in the lowest asset index tertile (SES) are more likely to fall behind in education (−0.0870*** for the Depression SEM, and −0.0871*** for the Anxiety SEM). Finally, adolescents who report more pro-social peers (Y-HAPP) are less likely to have consumed alcohol in the last 30 days (−0.0186*** for the Depression SEM, and −0.0190*** for the Anxiety SEM).

### 4.4. Limitations

This study is not without its limitations. The GAD-7 used has a reproduction error reported by several studies, in which the lowest response option to each item is “not at all sure” rather than the correct response “not at all”. This error potentially disrupts the psychometric properties that indicate the quality and effectiveness of the GAD-7 in identifying anxiety. One possible consequence is a bias towards an increased selection of this response option. Zorowitz et al suggest that “although this bias would probably have only a minor effect on individual scores and scale means, the effect of the error on the sensitivity of the scale in detecting clinical anxiety is unknown” [57]. Desmond et al. describe the non-linear and context specific relationship between social determinants and risky health behaviours. Our linear path analysis might not capture nuances between the identified social determinants and mental health outcomes [58].

Our study focuses on young people from a particular region who encounter a particular set of difficulties, we cannot assume these findings are uniformly generalisable across South Africa. In addition, given that we only use data from a single wave of the Asenze cohort study we undertook during adolescence, our analysis does not account for childhood predictors accounted for in earlier waves.

Data was collected before and during the COVID-19 pandemic and will have been influenced by the policies adopted by the South African government in this period, particularly in education, and the social and economic implications of the pandemic. This may have affected several of our social determinants, including family structures, living arrangements, employment and schooling.

## 5. Discussion

The key structural determinants of mental health in our sample were either being behind in education or NEET. These adolescents were significantly more likely to report poor mental health outcomes. Experiencing community violence had both a direct negative effect on adolescent mental health, and an indirect effect through reducing adolescents’ sense of school membership- illustrating the relationship between structural and proximal determinants. This corresponds with the literature showing that living in a safer environment in South Africa is positively related to better mental health outcomes [25], healthy peer relationships and a lower likelihood of adopting of risky behaviours for adolescents [36]. The indirect results also show that sex may be a significant modifier of the relationship between community violence exposure and mental health outcomes. The finding is similar to the limited evidence that shows that male and female adolescents may respond differently to exposure to community violence, although the direction of this relationship is unclear [26, 59].

Adolescent grant status and household assets were not significantly associated with mental health outcomes. While lack of household assets has been linked to increased odds for depression amongst South African adolescents [60], and cash grants have been associated with improving their mental health; our study area is relatively homogenous which could contribute to this lack of a significant effect. One exception is that adolescents who work or do something for money are likely to show more signs of anxiety.

In our sample, living with your biological mother, a sense of school belonging, and having prosocial peers are key protective proximal determinants against signs of depression and anxiety. Approximately 70% of adolescents in this cohort live with their biological mother, while only 28% live with their biological father. Multiple country-specific studies show that a non-intact family structure can negatively affect adolescent mental health [30]. However, in South Africa there is a trend of multi-generational households, and of children living with their biological mother rather than both biological parents [61]. Thus, the structure of families in South African households may have differing protective or harmful effects on the mental health of adolescents. For instance, secure attachments to mothers and support from extended family may be sufficient to bridge the health gaps between single-parent and two-parent households [62].

Adolescents form peer and school attachments in addition to their familial attachments [31]. A sense of school membership is negatively correlated with poor mental health outcomes. In addition, the greater the sense of belonging the adolescent felt to their school, the less likely they were to fall behind in their education and to have smoked cigarettes. This finding is significant and contributes to the limited evidence on the important role that psycho-social factors play on the health outcomes of adolescents in South Africa [33-35]. Similarly, having prosocial peers was protective of mental health, and reduced the likelihood of alcohol consumption. The finding supports the relationship described in our conceptual framework and found in global literature [38-40].

Adolescents in our sample who smoked in the prior 30 days were more likely to report being depressed. Common hypotheses for the relationship between smoking and depression include smoking as “self-medication” for depression, no causal relationship but rather common risks, and nicotine exposure as causing depression. A systematic review of the association between depression and smoking in adolescence found the relationship bidirectional but suggested that further research is needed to ascertain whether this association is causal [63].

Finally, female adolescents were more likely to report symptoms of depression and anxiety than their male peers. In socio-economically constrained environments, adolescent girls may face the brunt of challenges such as childbearing, dropping out of education to assist with care work, or participating in vulnerable occupations [17]. However, the gendered nature of the results may also be attributed to gender differences in the display of mental health challenges as captured by the GAD-7 and PHQ-9.

These results emphasize several potential points of intervention. The Lancet Psychiatry Commission on Youth Mental Health identifies the need to “increase community awareness, education, and advocacy, especially for prevention and the social and economic determinants of mental ill health” [2] as a key element of youth mental care. This need is reflected in the findings of our study. At the household and community level, the protective role of sense of school belonging and pro-social peers highlight the important role of the proximal social determinants in adolescent’s daily life on their mental health outcomes. Schools present a key opportunity to protect the mental health of children and adolescence as they are a point of connection between parents, youth, educators and the community, with existing infrastructure and networks and “are more accessible, and less stigmatised, than specialised mental health services” [64]. National policy in South Africa outlines the mental health services that should be available to learners within schools, however in actuality access to such services limited [65-67].

Our findings on the factors related to the structural determinants of poor mental health–specifically being behind in education or NEET and exposure to violence–require broader, systemic change to improve. Extensive, empirical evaluations of the South African schooling system have shown that it compares poorly to other countries, with below-average teacher content knowledge, high-levels of school drop-out before grade 12, insurmountable learning deficits and persistent inequalities in educational opportunities [68]. While the unemployment rate for youth aged between 15 and 24 was 60.7% in 2023 [12]. Community violence in the South Africa is so pervasive that it is often referred to as the one of the four major burdens of disease [69].

## 6. Conclusion

Our study sheds light on the social protective and risk factors that may influence adolescent mental health in under-resourced settings. Our findings indicate that living with the biological mother, the perception of family and peer support, school enrolment, and a positive sense of school membership may protect adolescent’s mental health. Conversely, exposure to community violence may be directly detrimental to adolescent mental health and indirectly harm mental health through the negation of a sense of school membership.

Considering the direct and indirect effects of social determinants on mental health highlights the value of looking at the adolescent’s context as a whole. The proximal social determinants show possible points of intervention close to the adolescent. While the structural determinants point to complex higher-level systematic issues which, if improved, could protect mental health in youth. Taken together, these findings suggest that effective interventions should extend beyond the traditional health system. If we limit our efforts to sector-specific single-outcome interventions, we may fail to address many of the broader social determinants of mental health. If decision-makers take a broader view of the determinants of mental health in adolescence, they can consider the opportunities that implementing interventions that span multiple development domains present. Our results suggest that the potential of school-based interventions and community-level violence reduction for preventing common mental health disorders in adolescence warrants investigation. Further, we suggest that such interventions may have positive outcomes for other domains beyond mental health. In prioritising common social determinants of challenges, we have the opportunity to improve people’s lives holistically. Further research is needed to establish causal links between social determinants of mental health in adolescents, as well as to explore the long-term impacts of early childhood experiences on adolescent mental well-being.

## Supplementary Material

Analytic dataset S1 Data. Supplementary material analytic dataset.

## Figures and Tables

**Table 1.** Literature to inform the conceptual framework.

**Structural Determinants**	**Socio- economic status** Mental health disorders and emotional distress may be triggered by challenging socio-economic conditions [17].	***Financial Shocks*:** Exogenous positive financial shocks—such as cash transfers—may have a positive impact on the mental health of adolescents [18, 19], through improved physical health, increased schooling and subsequent family support, and higher levels of consumption and leisure [ 19].
		***Educational Attainment*:** Poor educational conditions and attainment are a risk factor for mental disorders in children and adolescents [17, 20]. Increased expectation of future earning potential and resource command due to educational attainment has been linked to increased health improving behaviour, social skills and social integration [21]. The relationship between educational attainment and mental health may be attenuated by an unsupportive schooling environment [22]. There is some evidence that educational attainment mediates the relationship between poor parental socio-economic status and adverse mental health [20].
		***Employment status*:** Unemployment of the adolescent’s caregiver or other family members can negatively impact adolescent mental health [23] Studies measuring the impact of adolescent’s employment on their mental health tend to focus on out-of-school youth. Few studies investigate the impact of employment on the mental health of adolescents still in school, when we hypothesize may act as an additional stressor, or conversely may mediate the stress from socio-economic uncertainty and provide independence.
	**Community Exposure to Violence:** There is a close relationship between exposure to community violence, violence in schools and post-traumatic stress symptoms, aggression in adolescence and other mental health outcomes [24-26].	
	**Gender equity and gender norms:** **E**xternal and internalized, may impact the mental well-being of adolescents [27]. These norms pressure adolescents to conform to rigid gender roles and expectations into adulthood, limiting their freedoms, and potentially harming their psychological well-being [19, 28].	
Proximal Determinants	**Family structure:** plays a role in the mental health of adolescents [29]. Country-specific estimations found that non-intact family structures negatively influence adolescent mental health [30].	
	**Sense of School Membership/connectedness** is positively associated with adolescent mental well-being, mostly in high-income countries [31, 32], with limited evidence from South Africa [33-35]. In South Africa positive school connectedness buffers against negative mental health outcomes regardless of whether the respondent was an orphan [34].	
	**Hunger and food security:** accelerators poor adolescent mental health outcomes in a socio-economically disadvantaged South African setting [36]. Food insecurity elicits a complex array of stressors which may heighten psychological distress, anxiety and depression [37].	
	**Pro-social Peers:** Among young adults, peer support has been associated with improved self-esteem and coping skills, greater happiness and reductions in depression and anxieties [38]. Pro-social peers affects behavioural choices like risky sexual behaviour, smoking and alcohol consumption [39, 40].	
	**Risk and Health behaviour: Substance use** is associated with worse mental health outcomes among adolescents [41-43]. Cigarette use is much higher among individuals with serious mental illness compared to the population average [44].	

**Table 2.** Summary of the social determinants included in the analysis.

Social Determinant	Variable(s) in data
Demographic	
Sex	Adolescent sex (“female” or “male”); Caregiver sex (“female” or “male”);
Age	Age (measured continuously in years)
Structural Determinants	
**Employment and Education**	**Binary variable**: Caregiver reported whether household head is “unemployed” or “employed”. **Binary variable**: Adolescent reported if they were currently working. **Binary variable**: Adolescent reported if they were too old for their reported grade (“behind in education” or “not behind in education”). **Single binary variable**: Adolescent reported if they were not in any form of education, employment, or training (NEET).
**Household Socio-economic status**	**Binary variable:** An asset index was created based on the caregiver’s reporting of household asset ownership, and divided into tertiles. A binary variable is included in the analysis that is equal to one if adolescent’s household is in the poorest tertile (“Lowest Tertile” versus “Middle and upper Tertile”). **Binary variable:** adolescent is the beneficiary of any social grant.
**Conflict** Exposure to community violence (CVC)	**Scale:** Adolescents were asked the 12-item CDC Children’s Exposure to Community Violence scale [48] about the frequency of their exposure to violence in their neighbourhood and home. The scale ranges from 0 to 36, with a higher value indicating a higher exposure to violence.
**Gender** equity and roles	**Scale:** Adolescents were asked 15 questions about their perspectives on gender roles in the Adapted Gender Equitable Measurement (GEM) Scale [49]. The scale ranges from 0 to 12, with higher values indicating a more equitable perspective on gender roles.
Proximal determinants	
**Material Circumstances:** Household food security	**Binary variable:** Caregivers were asked “In the past 4 weeks, how often did you or any member of your household go to sleep hungry because of lack of food?” Adolescents were considered living in a food-secure household if their caregiver responded “never”.
**Material Circumstances:** Family structure	**Binary variable:**: residing with the biological mother; **Binary variable:** residing with the biological father, **Binary variable:** grandparent is the primary caregiver.
**Material Circumstances:** School Environment	**Scale:** Adolescents reported on their school environment in the 18-item Psychological Sense of School Membership (PSSM) [50]. A higher value indicates a higher sense of school connectedness.
**Material Circumstances:** Peers: Prosocial peers	**Scale:** Adolescents answered selected questions on pro-social peer behaviour, support from peers, and peer risk behaviour from the Youth Health and Prevention Project (Y-HAPP) [51]. The index ranges from 1 to 10, with a higher value indicating more pro-social peers.
**Risk and Health behaviour:** Smoking cigarettes	**Binary variable**: Adolescents self-reported whether they smoked cigarettes in the last 30 days or not.
**Risk and Health behaviour:** Alcohol consumption	**Binary variable:** Adolescents self-reported whether they drank alcohol in the last 30 days or not.

**Table 3.** Indirect relationships tested in our model, as derived from the literature.

Indirect relationship	Description
(a) Biological Mother in HH -> Grant recipient -> Depression/Anxiety (b) Biological Mother in HH -> Grant recipient -> Behind in Education -> Depression/Anxiety	The indirect effect having a biological mother in the household may have on adolescent’s mental health, through its effect on (a) the increased probability of being a grant recipient, as well as (b) the impact of grant recipience on educational outcomes.
(c) Adolescent is grant beneficiary->Behind in Education -> Depression/Anxiety	The indirect effect of grant recipience on mental health outcomes through (c) its role on adolescent educational outcomes.
(d) Adolescent is working->Behind in Education -> Depression/Anxiety	The indirect effect of a school-going adolescent being in employment on their mental health via the impact of adolescent employment on their (d) educational performance.
(e) Experience of community violence -> PSSM -> Depression/Anxiety Experience of community violence -> PSSM -> Behind in Education PSSM -> Behind in Education-> Depression/Anxiety (h) Experience of community violence -> PSSM -> Behind in Education -> Depression/Anxiety	The indirect effect of exposure to community violence on mental health outcomes through its impact on (e) school belonging (PSSM), and (f) the subsequent effect this might have on educational outcomes. We test whether the impact of exposure to community violence on educational outcomes is (g) mediated by a sense of school belonging (PSSM). While school belonging may have a direct impact on an adolescent’s mental health outcomes, we also test (h) the role of poor educational outcomes on this relationship.
(i) PSSM -> Smoked->Anxiety	Whether school belonging is related to the likelihood of smoking, and through this, mental health (i).
(j) Female -> Experience of community violence -> Anxiety	Whether sex is a potential moderator of the impact of community violence exposure on adolescent mental health (j).
(k) SES1 -> Behind in Education -> Anxiety	The indirect effect of an adolescent’s household socio-economic wealth status or asset index (SES) on their mental health via the impact on their (k) educational performance.
(l) Y-HAPP -> Drank alcohol -> Anxiety	The role of having positive peer role-models (Y-HAPP) on the likelihood of consuming alcohol, and whether this mediates some of the impact of alcohol consumption on mental health outcomes (l).

**Table 4.** Descriptive statistics of the study sample.

Category	Variable	Proportion or Mean (Count)
**Demographic Variables**	Age [Mean (Count)]	15.9 (1174)
	Female	0.5 (1174)
	Female Caregiver	0.95 (1138)
**Structural determinants: systems and opportunities**	Household head unemployed	0.6 (1133)
	Adolescent currently works or does something for money	0.15 (1170)
	Education: Adolescent too old for grade	0.21 (1169)
	Adolescent is NEET	0.03 (1169)
	Adolescent is grant recipient	0.85 (1135)
	Lowest SES	0.33 (1035)
	Index: Exposure to community violence (0 to 36) [Mean (Count)]	10.3 (1022)
	Index: Adapted Gender Equitable Measurement Scale (0 to 12) [Mean (Count)]	4.84 (986)
**Proximal determinants: circumstances of daily life**	Biological mother lives in household	0.71 (1138)
	Biological father lives in household	0.28 (1138)
	Indicated caregiver is grandparent	0.17 (1138)
	Adolescent’s household is food secure	0.82 (1134)
	Index: Psychological Sense of School Membership scale [Mean (Count)]	43.7 (1043)
	Index: Youth Health and Prevention Project selected questions on friends + peer pressure [Mean (Count)]	7.92 (980)
	Health and risk behaviour: adolescent reports smoking in the last 30 days	0.09 (1125)
	Health and risk behaviour: adolescent reports drinking alcohol in the last 30 days	0.12 (1123)

**Table 5.** Regression results: Direct effects of the social determinants on adolescent depression and anxiety.

Category	Variable	Depression (1)	Anxiety (2)
**Demographic Variables**	Age	−0.0266	−0.0314
		(0.0221)	(0.0215)
	Female	0.175***	0.107***
		(0.0411)	(0.0399)
	Female Caregiver	0.0364	0.0402
		(0.0926)	(0.0910)
**Structural determinants: systems and opportunities**	Household head unemployed	−0.00640	0.0181
		(0.0410)	(0.0403)
	Adolescent currently works or does something for money	0.0572	0.104**
		(0.0536)	(0.0523)
	Education: Adolescent too old for grade	0.0999**	0.0826*
		(0.0500)	(0.0488)
	Adolescent is grant recipient	0.00539	0.0442
		(0.0550)	(0.0537)
	Adolescent is NEET	0.418***	0.255**
		(0.114)	(0.111)
	SES: Lowest SES	−0.0265	−0.0611
		(0.0446)	(0.0442)
	Index: Exposure to community violence (0 to 36)	0.0229***	0.0197***
		(0.00338)	(0.00329)
	Index: Adapted Gender Equitable Measurement Scale (0 to 12)	0.00781	0.0160
		(0.0105)	(0.0102)
**Proximal determinants: circumstances of daily life**	Biological mother lives in household	−0.109**	−0.109**
		(0.0469)	(0.0460)
	Biological father lives in household	0.0368	0.00155
		(0.0432)	(0.0424)
	Indicated caregiver is grandparent	0.0157	−0.0814
		(0.0568)	(0.0558)
	Adolescent is food secure	0.0385	0.0471
		(0.0521)	(0.0510)
	Index: Psychological Sense of School Membership scale	−0.0214***	−0.0255***
		(0.00389)	(0.00375)
	Index: Youth Health and Prevention Project selected questions on friends + peer pressure	−0.0435***	−0.0210
		(0.0137)	(0.0132)
	Health and risk behaviour: adolescent reports smoking in the last 30 days	0.172**	0.0844
		(0.0720)	(0.0699)
	Health and risk behaviour: adolescent reports drinking alcohol in the last 30 days	−0.0150	0.00305
		(0.0608)	(0.0592)
	Observations	1,174	1,174

Standard errors in parentheses

*** p<0.01

** p<0.05

* p<0.1

**Table 6.** Indirect relationship between the social determinants and mental health outcomes.

	Indirect Relationship	Depression		Anxiety	
		Indirect effect	P-value	Indirect effect	P-value
a)	Biological Mother in HH -> Grant recipient -> Depression/Anxiety	0.0001593	0.922	0.0013066	0.491
b)	Biological Mother in HH -> Grant recipient -> Behind in Education -> Depression/Anxiety	−0.0000496	0.654	−0.0000405	0.661
c)	Adolescent is grant recipient->Behind in Education -> Depression/Anxiety	−0.001679	0.631	−0.0013702	0.639
d)	Adolescent is working->Behind in Education -> Depression/Anxiety	0.004068	0.295	0.0033214	0.324
e)	Experience of community violence -> PSSM -> Depression/Anxiety	0.0027243	0.001***	0.0032529	0.000***
f)	Experience of community violence -> PSSM -> Behind in Education -> Depression/Anxiety	0.0000758	0.126	0.0000639	0.165
g)	Experience of community violence -> PSSM -> Behind in Education	0.0007589	0.019**	0.0007736	0.018**
h)	PSSM -> Behind in Education->Depression/Anxiety	−0.0005949	0.102	−0.0005001	0.142
i)	PSSM -> Smoked-> Depression/Anxiety	−0.0010584	0.037**	−0.0005477	0.241
j)	Female -> Experience of community violence -> Depression/Anxiety	−0.0412881	0.000***	−0.0346667	0.000***
k)	SES1 -> Behind in Education -> Depression/Anxiety	0.0086911	0.092*	0.0071928	0.137
l)	Y-HAPP -> Drank alcohol -> Depression/Anxiety y	0.0002796	0.806	−0.000058	0.959

*** p<0.01

** p<0.05

* p<0.1

## Data Availability

Data is supplied in the supplementary material.
